# Modification of the Ladder Rung Walking Task—New Options for Analysis of Skilled Movements

**DOI:** 10.1155/2013/418627

**Published:** 2013-03-12

**Authors:** Iwa Antonow-Schlorke, Julia Ehrhardt, Marcel Knieling

**Affiliations:** Hans Berger Department of Neurology, Jena University Hospital, Friedrich Schiller University, 07747 Jena, Germany

## Abstract

Method sensitivity is critical for evaluation of poststroke motor function. Skilled walking was assessed in horizontal, upward, and downward rung ladder walking to compare the demands of the tasks and test sensitivity. The complete step sequence of a walk was subjected to analysis aimed at demonstrating the walking pattern, step sequence, step cycle, limb coordination, and limb interaction to complement the foot fault scoring system. Rats (males, *n* = 10) underwent unilateral photothrombotic lesion of the motor cortex of the forelimb and hind limb areas. Locomotion was video recorded before the insult and at postischemic days 7 and 28. Analysis of walking was performed frame-by-frame. Walking along the rung ladder revealed different results that were dependent on ladder inclination. Horizontal walking was found to discriminate lesion-related motor deficits in forelimb, whereas downward walking demonstrates hind limb use most sensitively. A more frequent use of the impaired forelimb that possibly supported poststroke motor learning in rats was shown. The present study provides a novel system for a detailed analysis of the complete walking sequence and will help to provide a better understanding of how rats deal with motor impairments.

## 1. Introduction

Stroke survivors commonly retain motor disabilities for years or even decades. Improvement of chronic poststroke motor dysfunction can be facilitated by special training [1, 2], medication [3, 4], and multisensory stimulation methods (for review, see [5, 6]). Moreover, recent evidence suggests that improvement of motor performance can be achieved over a prolonged time window [1, 2, 7–10]. In this context and for purposes of experimental studies, it is of special interest to have skilled motor tasks at one's disposal to evaluate postlesion motor performance in detail, particularly the level of impairment and functional recovery [11, 12].

In the present study, we modified the ladder rung walking test originally introduced by Metz and Whishaw [13] to analyse locomotion of rats across a horizontal rung ladder. In the above test, operations of all 4 paws are rated according to a qualitative score, and a mean value for each paw reflects the overall accuracy of paw use. Another option for locomotion assessment with the test represents calculating the percentage of errors in relation to the number of steps [13]. The ladder rung walking test has been shown to be sensitive to age-related motor deficits [13] and to different models of motor dysfunction [13–17]. We hypothesized that crossing an inclined rung ladder placed at an angle would give the test an added dimension of complication and would discern additional aspects of locomotion disabilities. In order to test our hypothesis, we analysed the ladder rung walking task in young adult rats for horizontal, upward, and downward walking directions. Rats were exposed to a unilateral photochemical lesion of the sensorimotor cortex of the forelimb and hind limb to assess postlesion motor performance. Furthermore, in modification to Metz and Whishaw [13], we analyzed the complete step sequence along the rung ladder. The complete step sequence also includes error-associated steps to facilitate studying the walking pattern.

Our results demonstrate distinct changes in locomotion of rats depending on the walking direction and indicate that walking along an inclined rung ladder is more complex than walking along a horizontal one. Moreover, inclined walking was associated with a more sensitive discrimination of postlesion motor disabilities in hind limb use. In the present study, we present an advanced analysis of skilled walking based on inspection of the complete stepping sequence along the whole distance of the rung ladder.

## 2. Methods

### 2.1. Subjects

Animal experiments were approved by the animal welfare commission of the Thuringian government. Ten young adult male Wistar rats weighing 280–300 g were used. Rats were raised and housed in groups of 5 animals under standard housing conditions (cages 34.7 × 55.0 × 18.5 cm, light/dark cycle 12/12 h, 22 ± 2°C) at the Institute of Laboratory Animal Science, Friedrich Schiller University, Jena. Water and food (standard pellets) were provided *ad libitum*.

### 2.2. Experimental Design

After a quick training of two trials along the horizontal ladder, rats were put through the walking tasks along the rung ladder in horizontal, upward, and downward positions for baseline values. Each task included five trials per animal. On the following day, rats underwent unilateral photothrombotic motor cortex lesion. Ladder rung walking tasks were repeated at post ischemic days (PI) 7 and PI 28. Here, rats completed the horizontal ladder first, followed by the upward and downward ladder rung walking tasks in a random order. For all the trials during the tasks, and on each day of testing, the rung positions were not changed to provide the same test conditions for all animals. In addition, the tests were scheduled from 09:00 to 12:00 hours. Finally, all behavioral tests were video recorded. Analysis of recording was performed by an investigator (JE) unaware of the experimental protocol.

### 2.3. Motor Cortex Lesion

Cortical photothrombotic lesion affecting the forelimb and hind limb motor areas was induced using an optic fiber bundle mounted on a cold light source (KL 1500 LCD, Schott, Mainz, Germany; 150 Watt) as previously described [18, 19]. In short, rats were anesthetized with 3.5% isoflurane (DeltaSelect GmbH, Germany) in 30% O_2_ and 70% N_2_O. Anaesthesia was maintained with 2.5% isoflurane. The head was then fixed on a stereotaxic apparatus. The scalp was opened to disclose the skull in an appropriate area, and the optic fiber bundle was positioned above the skull. A cover plate was placed on the skull to allow light transmission within a rectangular area limited by the coordinates Bregma −2.5 mm and 4.52 mm anterior-posterior and 0.5 mm and 4.5 mm lateral [20], but to exclude illumination of additional areas. A photosensitive dye Rose Bengal was injected intravenously (1.3 mg/100 g body weight in 10 mg/mL saline solution; Sigma-Aldrich, Taufkirchen, Germany), and the light (3200 K) was switched on for 20 min duration. Thereafter, anesthesia was concluded, and rats were allowed to recover. The lesions were induced on the left hemisphere of the brain as this affects the right body side and limbs.

Photothrombotic infarction was evaluated under light microscopy, and lesion size was estimated morphometrically as previously described [14, 18]. The photothrombotic occlusion resulted in focal damage of cerebral tissue that included the cortex and the corpus callosum. In two animals, the dorsal caudate putamen was also affected. The lesion size was 22.27 ± 8.52 mm^3^ (median 19.88 mm^3^).

### 2.4. Skilled Walking Apparatus

A rung walking apparatus was manufactured by in-house mechanics of the Jena University Hospital according to Metz and Whishaw [13, 21]. It consisted of two Plexiglas side walls linked by insertion of metal rungs (Ø 3 mm). In so doing, a floor was created that was 1.0 m in length and 19.0 cm in height with variable width that could be adjusted to fit the rat body size. In order to examine for higher test sensitivity, the rungs were placed at irregular intervals with rung-to-rung distances between 1.0 and 5.0 cm [13].

### 2.5. Modifications of Skilled Walking Task

In the present study, the angle of inclination of the rung ladder for both the upward and downwards walking tasks was 18°. This angle arose from the inclination of the rung ladder (length 1.0 m) to the upper edge of the slender wall in front of rat standard cages. Rats were supposed to walk from a neutral platform across the ladder to their home cages for horizontal and upward walking. For downward walking, rats reached a neutral platform next to their home cages and were placed therein immediately upon arrival.

### 2.6. Video Recording

Rats' walking was video recorded from a slight ventral view using a Panasonic Video high 3-CCD portable camera recording at 30 frames per second with a shutter speed set at 1/1000 s. Tapes were digitalized, and recordings were analyzed on a frame-by-frame basis using the software VirtualDub (version 1.9.11, freeware, Avery Lee).

### 2.7. Foot Fault Scoring

A qualitative analysis of skilled walking was performed using the foot fault scoring system [13, 21]. Foot placement and accuracy of the foot position on the rung was rated using a seven-category scale defined as follows: the midportion of the palm of the limb is placed on the rung with full weight support (*correct placement,* 6 points); the limb is placed on the rung with either wrist or digits of the forelimb or heel or toes of a hind limb (*partial placement,* 5 points); the limb aims for one rung but then is placed on another rung without touching the first one or, alternatively, the limb is placed on a rung but quickly repositioned (*correction,* 4 points); the limb is placed on a rung, but before it bears the weight, it is quickly lifted and placed on another rung (*replacement, *3 points); the limb is placed on a rung, slips off when weight bearing, but the animal is able to maintain balance and continue a coordinated gait (*slight slip,* 2 points); the limb is placed on the rung and slips off when weight bearing which leads to a fall (*deep slip*, 1 point); the limb completely misses the rung resulting in a fall (*total miss,* 0 points). The placement of all limbs was separately evaluated using frame-by-frame analysis of the video recordings. Five trials per rat were evaluated, and the scores were averaged for analysis [13, 21].

In order to analyze the complete step sequence, analysis of the foot fault scoring system was modified as follows: starting with the limb that began the walk, consecutive steps were all estimated. Therefore, stops and steps that were associated with stepping errors were included into analysis.

### 2.8. Errors in Foot Placement Accuracy

A quantitative analysis of skilled walking was performed using the mean ratio of errors per step [13, 21]. Inadequate limb placement that involved missing the rung or slipping off (rated with 0, 1, or 2 points, see above) was defined as an error. For each crossing, the number of errors and the number of steps were counted separately for each limb and represented as a ratio of errors. The ratio of errors per step was averaged for five trials [13, 21].

### 2.9. Walking Pattern Analysis

**Figure 1:**
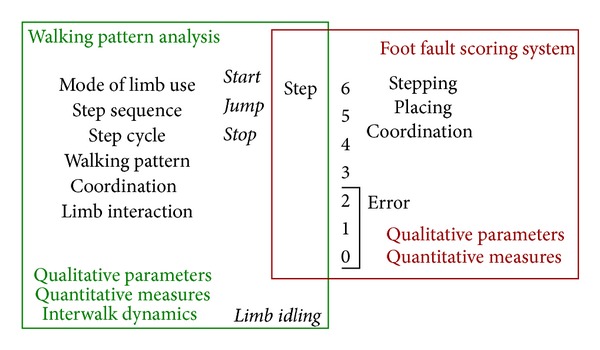
Proposed system of comprehensive analysis of skilled walking. Analysis of steps represents a central part of the system. Foot placement and accuracy is rated, and foot placement errors are counted using the foot fault scoring system (red box) introduced by Metz and Whishaw [13]. Step analysis is complemented by observance of the complete step sequence of a walk and by introduction of new categories for different modes of limb use (green box). Beginning the analysis with the limb that starts walking (*start*) and tracking consecutive steps can show the detailed sequence of steps and step cycles and enables the recognition of *stops* and limb *idling. *Dynamics in limb operation markedly differ between *steps* and *jumps*. Modes of limb use contribute to walking pattern and allow conclusion regarding limb coordination and limb interaction (green box).

In order to question the natural walking pattern, the complete stepping sequence along the whole distance of the rung ladder was inspected. To evaluate the walking pattern, step sequence, and mode of limb use, the following categories were used: *Start* marks the limb that starts walking; *Jump* marks a limb that does the hopping move; *Stop* marks an interim stop and thereby the rat stops the walk for a short moment;* idle* the limb is not used in the actual stepping cycle.  Figure 1 summarizes the new categories of the walking pattern analysis and illustrates the complementation of foot fault scoring that were intended for a more comprehensive analysis of walking (Figure 1).

### 2.10. Statistical Analysis

Statistic analyses were performed using the software package SPSS (version 16.0). The Friedman-Test was used as a global test for nonparametric data to obtain differences during the postischemic time course. Subsequently, the Wilcoxon Rank Test for related data was used to compare the mean value of the postischemic time course to baseline (two-tailed) closing with Bonferroni-Holm Correction to correct the time-dependent *α*-error. For comparison of values of the foot fault scoring system and error rate between the different experimental tasks, the Wilcoxon-Rank-Test for related data was used (two-tailed) closing with Bonferroni-Holm-Correction to correct the *α*-error due to multiple testing. Correlations between stepping performance (foot fault score) and the respective error rate were estimated using the Pearson coefficient and the Spearman coefficient to describe linear or monotone correlations (two-tailed), respectively. A significance level of *P* < 0.05 was chosen. Data are presented as mean ± standard error of the mean (SEM).

## 3. Results

### 3.1. Placement and Accuracy of Limb Use (Qualitative Analysis)

All rats were able to resolve each one of the different ladder rung walking tasks. Foot placement and accuracy reflected photothrombotic motor cortex lesion and differed according to the inclination of the ladder.

#### 3.1.1. Horizontal Ladder Rung Walking

At baseline, the mean score values ranged from 5.44 ± 0.14 to 5.64 ± 0.11 for all paws (Table 1). Photothrombosis of the motor cortex induced significant deficits of the impaired limb at postischemic day 7 that were reflected by a drop in rating values of 19.2% and a 9.4% of the right fore and hind limb, respectively (*P* < 0.01, Table 1). At postischemic day 28, however, score values for both right limbs were increased in comparison to postischemic day 7 (*P* < 0.05, Table 1). Here, motor deficits of the right forelimb still existed compared to baseline (*P* < 0.01, Table 1) but the right hind limb had recovered (Table 1). The use of the ipsilateral limbs was rated as constant during the experimental period (Table 1).

#### 3.1.2. Upward Ladder Rung Walking

At baseline, limb use was rated between 5.22 ± 0.12 and 5.79 ± 0.11 points (Table 1). At postischemic day 7, motor cortex injury resulted in reduced scores for the impaired forelimb and hind limb by 24.3% and 13.6%, respectively (*P* < 0.01, Table 1). Until postischemic day 28, the use of both right limbs had improved (*P* < 0.05, Table 1). Here, the right forelimb achieved rating values similar to baseline (Table 1), but impairments of the right hind limb remained (*P* < 0.01, Table 1). Score values for the ipsilateral limbs did not change after injury (Table 1).

#### 3.1.3. Downward Ladder Rung Walking

At baseline, the mean score values for all paws ranged between 5.11 ± 0.14 and 5.65 ± 0.21 (Table 1). Ratings for the impaired forelimb and hind limb were decreased by 29.2% and 18.8%, respectively, at postischemic day 7 (*P* < 0.01, Table 1). At postischemic day 28, although score values for both impaired limbs were found increased in comparison to postischemic day 7 (*P* < 0.01, Table 1), hind limb use did not fully recover (*P* < 0.01, Table 1). Ratings for both intact limbs were similar to baseline after injury (Table 1). 

#### 3.1.4. Comparison of Ladder Rung Walking Tasks

**Figure 2:**
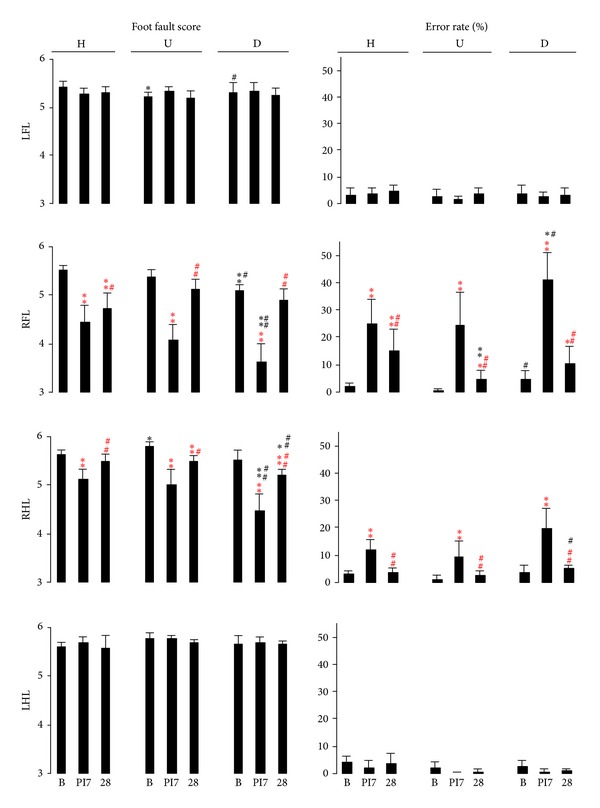
Limb placement and accuracy in inclined ladder rung walking. Horizontal (zero inclination), upward, and downward walking across the rung ladder were analyzed using qualitative ratings for limb placement and accuracy (foot fault score), and quantitative analysis (error rate in stepping), evaluated for each limb before and after focal photothrombotic lesion undertaken in the respective areas of the motor cortex of both right forelimb and hind limb. Lesion-related motor deficits of the hind limb were most sensitively detected in inclined walking. H: horizontal ladder, U: upward ladder, D: downward ladder, B: baseline, PI 7: postischemic day 7, PI 28: postischemic day 28, LFL: left forelimb, RFL: right forelimb, RHL: right hind limb, LHL: left hind limb.  ^one  red  stars^ *P* < 0.05,  ^two  red  stars^ *P* < 0.01 versus baseline,  ^red  #^ *P* < 0.05,  ^red  ##^ *P* < 0.01 versus PI 7; *P < 0.05, **P < 0.01 versus H, ^#^ *P* < 0.05, ^##^ *P* < 0.01 versus U.

At baseline, slight differences in score values were elicited by inclination of the rung ladder in comparison to the horizontal walk: along the upward ladder rung task, left forelimb use was rated lower by 4.0% (*P* < 0.05); however, the use of the right hind limb was rated higher by 2.6% (*P* < 0.05; Figure 2). At postischemic day 7 along the downward ladder rung, score values of the impaired forelimb were reduced by 18.8% and 11.3% in comparison to horizontal walking and upward walking, respectively (*P* < 0.01, Figure 2). In association, ratings of the impaired hind limb were reduced by 12.1% and 10.2% compared to horizontal walking and upward walking, respectively (*P* < 0.01, Figure 2). At postischemic day 28, score values of the impaired hind limb for downward rung ladder walking were found to be 7.0% less versus the scores given for each of the horizontal walking and the upward walking (*P* < 0.05, Figure 2).

### 3.2. Errors in Foot Placement (Quantitative Analysis)

The measure of errors in foot placement was sensitive to motor cortex lesion and to inclination in rung ladder walking.

#### 3.2.1. Horizontal Ladder Rung Walking

At baseline, the mean percentage of errors ranged between 2.10 ± 1.68% and 4.05 ± 2.95% for all paws (Table 1). Error rates of the impaired forelimb and hind limb rose by 11.9-fold and 3.7-fold, respectively, at postischemic day 7 (*P* < 0.01, Table 1). At postischemic day 28, error rates of both impaired limbs had decreased in comparison to postischemic day 7 (*P* < 0.01, Table 1), and the hind limb had recovered. In contrast, the error rate of the forelimb still had the 7.0-fold baseline value at postischemic day 7 (*P* < 0.01, Table 1). Photothrombotic lesion did not change the error rate of both intact limbs (Table 1).

#### 3.2.2. Upward Ladder Rung Walking

 At baseline, individual paws showed 0.65 ± 1.16% to 2.77 ± 3.07% errors in walking (Table 1). Motor cortex injury enhanced the error rates of the impaired forelimb and hind limb to 37.7-fold and 7.2-fold values, respectively, at postischemic day 7 (*P* < 0.01, Table 1). At postischemic day 28, error rates of impaired right limbs had dropped compared to postischemic day 7 (*P* < 0.01, Table 1). Thereby, the forelimb still showed a 7.3-fold higher error rate compared to baseline (*P* < 0.01, Table 1) but the hind limb did recover (Table 1). Error rates for both intact limbs were identical during the experiment (Table 1).

#### 3.2.3. Downward Ladder Rung Walking

 At baseline, 2.70 ± 2.70% to 4.86 ± 3.40% of all steps were found to fail (Table 1). Photothrombotic lesion caused a rise in error rates of the impaired forelimb and hind limb to 8.4-fold and 5.2-fold, respectively, at postischemic day 7 (*P* < 0.01, Table 1). At postischemic day 28, error rates for both impaired limbs were reduced compared to postischemic day 7 (*P* < 0.01, Table 1). Thereby, the error rate of the forelimb was 2.2-fold of baseline values (*P* < 0.05), but the hind limb already operated with a normal error rate (Table 1). Error rates of the intact limbs were stable after injury (Table 1).

#### 3.2.4. Comparison of Ladder Rung Walking Tasks

 At baseline, there were differences in error rates for the right forelimb between the inclined rung ladder walking tasks (Figure 2). At postischemic day 7, the impaired forelimb produced a 1.6-fold higher error rate crossing the downward rung ladder than crossing the horizontal one (*P* < 0.05), and which also differed from the rate calculated for upward rung ladder walking (*P* < 0.05, Figure 2). At postischemic day 28, the impaired forelimb showed a 3.1-fold lower error rate in upward walking compared to horizontal walking (*P* < 0.01, Figure 2). The impaired hind limb showed a similar error rate for upward walking compared to horizontal walking, whereas a 2.0-fold higher number of errors occurred for downward walking (*P* < 0.05, Figure 2).

### 3.3. Correlation between Accuracy of Limb Use and Errors in Foot Placement

The rating of limb accuracy determined out from the foot fault score and the number of errors in limb use were overall negatively related. A high linear or monotone correlation was shown for horizontal and downward ladder rung walking at baseline (Table 2) as well as for horizontal, upward, and downward walking at postischemic days 7 and 28 (Table 2).

### 3.4. Walking Pattern Analysis

#### 3.4.1. Jumps

**Figure 3:**
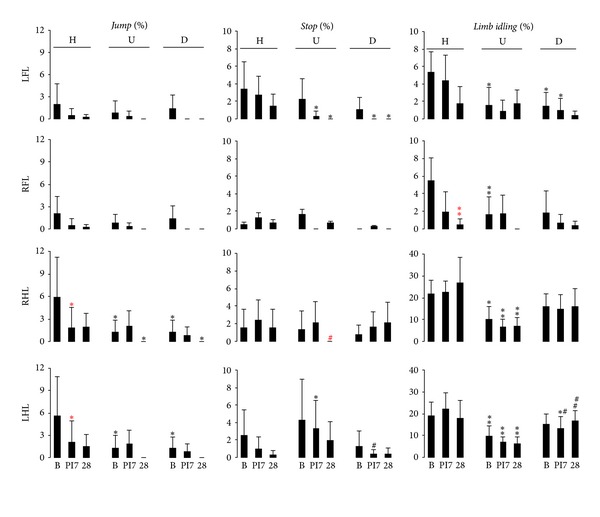
Modes of limb use in inclined rung ladder walking. Frequency of occurrence of the categories for limb use: *jumps, stops, *and* limb idling* were analyzed for each limb in horizontal (zero inclination), upward, and downward walking across the rung ladder before and after photothrombotic lesion. Lesion was performed in the respective areas of the motor cortex of both the right forelimb and hind limb. In horizontal walking, photothrombosis resulted in an increased use of the impaired forelimb, and modes of limb use differed between the tasks. H: horizontal ladder, U: upward ladder, D: downward ladder, B: baseline, PI 7: postischemic day 7, PI 28: postischemic day 28, LFL: left forelimb, RFL: right forelimb, RHL: right hind limb, LHL: left hind limb.  ^one  red  star^ *P* < 0.05,  ^two  red  stars^ *P* < 0.01versus baseline,  ^red  #^ *P* < 0.05 versus PI 7; *P < 0.05, **P < 0.01 versus H, ^#^ *P* < 0.05, ^##^ *P* < 0.01 versus U.

While crossing the horizontal ladder, rats executed jumps using the forelimbs for approximately 2% and the hind limbs for 6% of all the steps, respectively (Figure 3). Here, the forelimbs and the hind limbs operated synchronously. After photothrombotic lesion, the use of hind limbs for jumps was decreased (*P* < 0.05; Figure 3). In upward and downward ladder rung walking, the limb use synchrony for jumping was evident, but the frequency of hind limb jumping was significantly reduced compared to horizontal ladder rung walking (*P* < 0.05; Figure 3).

#### 3.4.2. Stops

The analysis of the complete sequence of walking steps across the whole ladder proved beneficial for identification of stopping as a further mode of limb use. Rats generally paused for very short moments whilst crossing the horizontal rung ladder. Here the short pauses occurred at a rate between 0.5% and 3.4% that did not change after injury (Figure 3). Whilst crossing the upward and downward rung ladder, healthy rats stopped walking at a similar frequency (Figure 3). Photothrombotic lesion particularly diminished the frequency of stops following impaired hind limb use in upward rung ladder walking at postischemic day 28 (Figure 3). In comparison to horizontal rung ladder walking, photothrombotic lesion reduced the frequency of stops of the intact forelimb in both upward and downward rung ladder walking but transiently increased stop frequency of the intact hind limb in upward walking (Figure 3).

#### 3.4.3. Limb Idleness

Analysis of the complete step sequence across the whole ladder allowed the identification of limb idling as an additional mode of limb use. Crossing the horizontal rung ladder, healthy rats on occasion did not use all four limbs for completing a step cycle. Thereby, rats used the forelimbs and hind limbs in about 95% and 80% of all the step cycles, respectively. In other words, in about 5% of the step cycles, the forelimbs were idle, and for 20% of step cycles the hind limbs were not used (Figure 3). The forelimbs and hind limbs operated almost synchronously here. Photothrombotic lesion led to a more frequent use of the impaired forelimb at postischemic day 28 (*P* < 0.01; Figure 3). In upward and downward ladder rung walking, limb *idling* was seen less often than in horizontal walking (*P* < 0.05), and cerebral injury had no additional effect (Figure 3).

## 4. Discussion

All rats were able to cross rung ladders installed both in a horizontal or an inclined position. At baseline, slight differences in foot placement accuracy and in errors per step indicated different requirements for rats' motor performance with respect to inclined rung ladder walking compared to horizontal rung ladder walking. Upward and downward rung ladder walking were associated with a sensitive detection of lesion-induced motor deficits of the affected hind limb. Analysis of the complete walking sequence proved advantageous for identification of modes of limb use in ladder rung walking, such as *jumps, stops,* and limb *idling*, thereby providing a comprehensive walking pattern. In horizontal walking, forelimbs and hind limbs did not participate in about 5% and 20% of the step cycles, respectively. *Jumps *and *stops* were observed at a relatively low rate. It was notable that rats responded to rung ladder inclination with subtle changes in the frequency of *jumps, stops,* and limb *idling*. Photothrombotic lesion reduced the frequency of *jumps* but increased the frequency of participation in step cycles of the affected forelimb.

The horizontal rung ladder walking task with an irregular rung pattern has previously been described as a sensitive test to evaluate both forelimb and hind limb use in rats after lesions of the motor system [13]. Our data show postlesion deficits of the impaired forelimb and hind limb after unilateral photothrombotic lesion as previously reported [14, 18]. Motor impairments, particularly in forelimb use, were reflected by a reduced foot fault score and an enhanced error rate that was sustained for a period of four weeks. It has often been reported that deficits in motor performance of the forelimbs predominate in experimentally evoked motor disabilities [22–26].

Testing rats for upward and downward ladder rung walking is a valid task. It mimics daily demands of living in an urban environment. Further, rung ladders are often used to enrich the cage environment for housing of laboratory rats. An inclined ladder was already used to evaluate climbing following a traumatic spinal cord injury in rats [27]. However, the nature of ladder rung walking tested herein has to our knowledge not been published to date.

Interestingly, our data clearly demonstrate that evaluation of motor deficits in rung ladder walking yields results correlating with the positioning of the rung ladder. Thus, inclining the rung ladder for upward walking revealed a need for a higher level of motor performance for forelimb and hind limb use compared to horizontal walking, reflected by higher foot placement scores together with stable error rates at baseline. Accordingly, upward walking on a rung ladder represents a more complex motor task than horizontal walking. Here, the additional dimension represented by gravity demands more load on hind limbs to propel the body upwards, and at the same time, more load is required in the forelimbs for maintaining balance and hold when hind limbs are being moved to a new rung. Our data show that healthy rats are partly able to improve motor performance for upward walking in response to the task. After unilateral lesion, long-lasting deficits of the affected forelimb were apparent via the increased error rate, whereas long-lasting deficits for the hind limbs were reflected by reduced foot placement scores. The motor deficits shown in upward walking clearly indicate that photothrombotic lesion has a strong influence on the accuracy of limb placement for both affected fore- and hind limbs, interlimb coordination, and balance.

Although healthy rats cope with walking downwards on the rung ladder, they show specific deficits in hind limb use. Crossing the ladder rung downwards accounts for a high load to the forelimbs in forward movement with challenging requirements in spatial cognition, limb coordination, weight bearing, and balance. During downward walking, long-lasting lesion-related deficits of the affected fore- and hind limbs were revealed with high sensitivity by means of scoring limb use and error rate. Clearly, rats suffer from motor impairments of both forelimb and hind limbs that point to problems in foot placement accuracy, interlimb coordination, and balance. Furthermore, the present findings show downward walking to elicit motor dysfunction in forelimb and hind limb use that exceed the level of deficits discovered for both horizontal and upward walking. These data underline the extraordinary demands of downward locomotion in the current study.

Since the irregular rung pattern was not changed during the experimental period, rats should have internalized this rung pattern during the repetitive trials [28]. Therefore, the impaired poststroke motor performance in downward walking here points to the actual limit of recovery of the motor system.

The close correlations between motor performances in foot placement and respective error rates shown here underline both the foot fault score and the error rate as valid parameters of skilled motor impairments as stated before [13]. However, under certain experimental conditions, correlation did not reach significance for individual limbs. Further, despite significant correlation, for example, for upward walking, long-lasting deficits in forelimb use were not reflected in foot placement score. Similarly, long-lasting deficits in hind limb use could not be detected by error rate. Accordingly, we and others might have overlooked valuable information in studies in which the observation was restricted either to a single category of foot placement [26] or to quantitative movement analysis [18]. As indicated by the compelling results obtained in the current study, we recommend assessment of both qualitative and quantitative parameters for walking analysis in order to fully exploit data resulting from the rung ladder test.

The high sensitivity of inclined rung ladder walking could facilitate the detection and observation of motor impairments in animal experiments. Because age-related differences in skilled walking of rats have previously been reported [13], inclined rung ladder walking promises to be an additional attractive tool to investigate skilled walking during aging. Otherwise, successful testing also requires the animals to be able to traverse the ladder [13]. In the current study, the induced lesions were focal ones, and rats had just turned into adulthood. Here, all rats performed every task well, and cerebral injury did not limit test practicability.

Moreover, it is conceivable that test sensitivity is altered by changing the demands in upward and downward walking by means of respective variation in the angle of ladder inclination.

The particular modes of limb use for rung ladder walking referred to as *jumps, stops,* and limb* idling* are analysed herein for the first time. In contrast to the natural safe walking pattern realized by consecutive step cycles to which all four limbs contribute [13, 29], our findings clearly demonstrate that rats perform walking along a rung ladder in different ways. Although all four limbs take part in the majority of step cycles, our data show that hind limbs remain *idle* at a surprising rate during horizontal ladder rang walking. Here, rats use forelimbs to cross rungs in consecutive steps, whilst the hind limbs move over many rungs at major intervals. Interestingly, the forelimbs also remained *idle*, although at a much lower rate. Because animals explore their way forward using their whiskers and forelimbs, it is reasonable to pronounce that forelimbs are more often used than hind limbs. Further, *jumps *and *stops* have been observed as infrequent, but consistent modes of limb use. *Jumps* demand great skill which accelerates forward locomotion. Although we cannot currently clarify the significance of the *stop *mode, it most likely reflects short interruptions during walking for orientation purposes. Further, locomotion and associated motor learning aim towards a high efficacy of motor performance [30, 31]. Thus, the walking pattern on the horizontal rung ladder has to be regarded as spontaneous and highly effective locomotion resulting from adjustments of the motor system to the actual task. Consequently, and as shown in the present study, healthy rats alter their mode of limb use depending on the position of the rung ladder. Thus, the frequency of *jumps *and limb *idleness *was adjusted whilst walking across an inclined ladder, since *jumps* during upward or downward walking are associated with a higher risk of rung failure and fall for the rat. Our data convincingly suggest that healthy rats are able to estimate their motor competence and avoid high-risk conduct. Correspondingly, a diminished rate of limb *idleness* indicates an increased rate of limb use or *vice versa *suggesting that inclined walking will be better mastered with an enhanced limb coordination than required for horizontal walking. Taken together, the reported modes for limb use in rung ladder walking demonstrate the ability of healthy rats to adjust their walking pattern to the demands of the given task. Therefore, our data clearly indicate *jumps *and limb *idleness/idling *as being sensitive modes of limb use for rung ladder inclination.

Surprisingly, our findings show that rats do not rest the impaired forelimb postlesion. In contrast, the impaired forelimb more frequently participated in performing step cycles. This suggests an urgent need for closer interlimb coordination to resolve the actual task, at least in the observed post-ischemia time period. Although this increase in the rate of limb use seems to be slight, it may contribute to postischemic improvement of motor function, reflected in the respective foot placement scoring and error rates. Thus, increased participation of the impaired limb in coordinated locomotion seems to be needed for effective poststroke motor learning. Interestingly, the concept of forced use of impaired extremities in stroke patients has become a successful mode of therapy [1, 2, 32].

*Jumps, stops,* and limb *idling* seem to represent optional actions for limb use along the rung ladder. Analysis of the frequency of occurrence allows insights in the walking pattern, step sequence, step cycle, mode of limb use, and limb coordination. Thus, the new categories substantially contribute to walking analysis and complement the foot fault scoring system. Moreover, discovery of the categories *stops* and limb *idling* herein resulted from a careful analysis of the complete sequence of steps.

We propose a novel system for comprehensive analysis of skilled walking (Figure 1) based on the foot fault scoring system [13] complemented with an analysis of the complete stepping sequence along the whole distance of the rung ladder. The new categories of limb use introduced here demonstrate several dynamic aspects of walking. The new category *start* marks the limb that starts the locomotion and documents the sequence of steps. *Idling* represents a counterpart to an actual step, and *jump* characterizes a counterpart to a step that refers to dynamics in limb operation. These modes of limb use contribute to walking pattern and allow conclusions regarding limb coordination and limb interaction. Thus, combining these different parameters for the analysis adds a new dimension to the evaluation of skilled walking. Hence, the synergistic effects between foot placement scoring and walking pattern analysis provide further options for a detailed analysis (Figure 1). The proposed system has proven its worth in the present study and promises to upgrade locomotion analysis. Using the ladder rung walking task in mice [33], the herein proposed novel analysis system could easily be applied to mouse locomotion analysis.

## 5. Conclusions

The present study established modifications of the horizontal ladder rung walking test that increase the demands of the task and enhance test sensitivity. New aspects of walking were included into analysis that allow evaluation of walking pattern, step sequence, step cycle, mode of limb use, limb coordination, and limb interaction and, hence, complement the foot fault scoring system. Horizontal walking showed lesion-related motor deficits in forelimb, and downward walking in hind limb use most sensitively. The analysis of subsequent steps proved beneficial for identification of infrequent modes of limb use that were optionally executed, and reflected task requirements. The ischemia-related increase in participation of the impaired forelimb in performing a step cycle found here, suggests limb training as an inherent principle of the motor system to optimize postlesion motor learning in rats. We believe the proposed novel system of walking analysis to be an innovative approach that will advance our understanding of motor behavior and motor learning.

## Figures and Tables

**Table 1:** Effect of cerebral ischemia on ladder rung walking in rats.

		Foot fault score						Error rate (% of steps)					
		B		PI 7		PI 28		**B**		PI 7		PI 28	
		Mean ± SEM	Median	Mean ± SEM	Median	Mean ± SEM	Median	Mean ± SEM	Median	Mean ± SEM	Median	Mean ± SEM	Median
Horizontal	LFL	5.44 ± 0.14	5.47	5.28 ± 0.16	5.30	5.30 ± 0.15	5.27	3.28 ± 2.79	3.72	3.42 ± 2.59	2.57	4.73 ± 2.72	3.89
	RFL	5.52 ± 0.13	5.49	4.46 ± 0.35	4.58	4.73 ± 0.34	4.90	2.10 ± 1.68	2.53	25.13 ± 9.31	23.81	14.83 ± 8.57	10.26
	RHL	5.64 ± 0.11	5.63	5.11 ± 0.25	5.10	5.50 ± 0.16	5.54	3.25 ± 1.56	3.03	12.01 ± 3.87	11.60	3.80 ± 1.80	3.13
	LHL	5.60 ± 0.11	5.57	5.68 ± 0.14	5.74	5.58 ± 0.29	5.74	4.05 ± 2.95	3.34	2.33 ± 2.79	1.61	3.57 ± 4.28	0.00

Up	LFL	5.22 ± 0.12	5.25	5.33 ± 0.12	5.30	5.18 ± 0.17	5.14	2.77 ± 3.07	0.00	1.34 ± 1.88	0.00	3.88 ± 2.50	3.72
	RFL	5.39 ± 0.16	5.46	4.08 ± 0.32	4.10	5.11 ± 0.25	5.25	0.65 ± 1.16	0.00	24.61 ± 12.20	30.52	4.75 ± 3.65	3.51
	RHL	5.79 ± 0.11	5.79	5.00 ± 0.35	5.02	5.50 ± 0.14	5.47	1.29 ± 1.72	0.00	9.26 ± 6.38	9.13	2.55 ± 2.04	3.57
	LHL	5.76 ± 0.16	5.77	5.78 ± 0.08	5.79	5.70 ± 0.08	5.69	1.97 ± 2.62	0.00	0.10 ± 0.18	0.00	0.72 ± 1.14	0.00

Down	LFL	5.32 ± 0.23	5.40	5.34 ± 0.19	5.42	5.25 ± 0.17	5.25	3.61 ± 3.67	1.67	2.41 ± 2.41	1.56	3.02 ± 3.02	1.72
	RFL	5.11 ± 0.14	5.07	3.62 ± 0.40	3.68	4.88 ± 0.26	4.92	4.86 ± 3.40	5.03	40.84 ± 10.27	40.78	10.60 ± 6.47	12.02
	RHL	5.53 ± 0.22	5.47	4.49 ± 0.37	4.46	5.21 ± 0.14	5.27	3.77 ± 3.03	3.77	19.57 ± 7.85	20.00	5.16 ± 1.70	4.17
	LHL	5.65 ± 0.21	5.72	5.69 ± 0.13	5.69	5.65 ± 0.08	5.64	2.70 ± 2.70	1.79	0.76 ± 1.21	0.00	0.83 ± 1.34	0.00

RFL: right forelimb, LFL: left forelimb, RHL: right hind limb, LHL: left hind limb, B: baseline, PI 7: post-ischemic day 7, PI 28: post-ischemic day 28.

Median values are given to show variability of data.

**Table 2:** Correlation between foot fault score and error rate.

		Horizontal			Up			Down		
		B	PI 7	PI 28	B	PI 7	PI 28	B	PI 7	PI 28
**LFL**	Pearson	**−0.637** (0.048)	−0.218 (0.546)	−0.622 (0.055)	−0.331 (0.384)	−0.594 (0.070)	−0.506 (0.136)	**−0.904** (0.000)	**−0.841** (0.002)	**−0.748** (0.013)
	Spearman	−0.427 (0.219)	−0.438 (0.205)	−0.541 (0.106)	−0.220 (0.569)	−0.551 (0.099)	−0.464 (0.176)	**−0.888** (0.001)	**−0.775** (0.009)	**−0.636** (0.048)
**RFL**	Pearson	**−0.690** (0.027)	**−0.975** (0.000)	**−0.985** (0.000)	−0.721 (0.280)	**−0.877** (0.001)	**−0.819** (0.004)	**−0.639** (0.047)	**−0.962** (0.000)	**−0.885** (0.001)
	Spearman	**−0.638** (0.047)	**−0.954** (0.000)	**−0.976** (0.000)	−0.550 (0.125)	**−0.821** (0.004)	**−0.804** (0.005)	**−0.816** (0.004)	**−0.818** (0.004)	**−0.900** (0.000)
**RHL**	Pearson	−0.614 (0.059)	**−0.867** (0.001)	**−0.833** (0.003)	−0.612 (0.080)	**−0.932** (0.000)	**−0.731** (0.016)	**−0.853** (0.002)	**−0.964** (0.000)	**−0.768** (0.009)
	Spearman	−0.505 (0.137)	**−0.833** (0.003)	−0.500 (0.141)	−0.611 (0.080)	**−0.884** (0.001)	−0.625 (0.053)	**−0.846** (0.002)	**−0.973** (0.000)	**−0.686** (0.029)
**LHL**	Pearson	**−0.883** (0.001)	**−0.718** (0.019)	**−0.887** (0.001)	**−0.763** (0.017)	n.e. —	**−0.789** (0.007)	**−0.913** (0.000)	**−0.737** (0.015)	−0.412 (0.237)
	Spearman	**−0.914** (0.000)	**−0.683** (0.029)	**−0.792** (0.006)	**−0.686** (0.041)	n.e. —	−0.083 (0.820)	**−0.921** (0.000)	**−0.683** (0.029)	−0.305 (0.392)

Pearson correlation coefficient and Spearman correlation coefficient are given with the level of significance in parentheses. Significant correlations are highlighted (*P* < 0.05).

n.e. not evident because one of the variables is constant (error rate = 0).

RFL: right forelimb, LFL: left forelimb, RHL: right hind limb, LHL: left hind limb, B: baseline, PI 7: post-ischemic day 7, PI 28: post-ischemic day 28.
